# Investigating the impact of poverty on colonization and infection with drug-resistant organisms in humans: a systematic review

**DOI:** 10.1186/s40249-018-0459-7

**Published:** 2018-08-17

**Authors:** Vivian Alividza, Victor Mariano, Raheelah Ahmad, Esmita Charani, Timothy M. Rawson, Alison H. Holmes, Enrique Castro-Sánchez

**Affiliations:** 1 NIHR Health Protection Research Unit in Healthcare Associated Infection and Antimicrobial Resistance, Imperial College London, London, UK; 2 Health Group, Management Department, Imperial College Business School, Exhibition Road, London, UK

**Keywords:** Poverty, Antimicrobial stewardship, Drug resistance

## Abstract

**Background:**

Poverty increases the risk of contracting infectious diseases and therefore exposure to antibiotics. Yet there is lacking evidence on the relationship between income and non-income dimensions of poverty and antimicrobial resistance. Investigating such relationship would strengthen antimicrobial stewardship interventions.

**Methods:**

A systematic review was conducted following Preferred Reporting Items for Systematic Reviews and Meta-Analyses (PRISMA) guidelines. PubMed, Ovid, MEDLINE, EMBASE, Scopus, CINAHL, PsychINFO, EBSCO, HMIC, and Web of Science databases were searched in October 2016. Prospective and retrospective studies reporting on income or non-income dimensions of poverty and their influence on colonisation or infection with antimicrobial-resistant organisms were retrieved. Study quality was assessed with the Integrated quality criteria for review of multiple study designs (ICROMS) tool.

**Results:**

Nineteen articles were reviewed. Crowding and homelessness were associated with antimicrobial resistance in community and hospital patients. In high-income countries, low income was associated with *Streptococcus pneumoniae* and *Acinetobacter baumannii* resistance and a seven-fold higher infection rate. In low-income countries the findings on this relation were contradictory. Lack of education was linked to resistant *S. pneumoniae* and *Escherichia coli*. Two papers explored the relation between water and sanitation and antimicrobial resistance in low-income settings.

**Conclusions:**

Despite methodological limitations, the results suggest that addressing social determinants of poverty worldwide remains a crucial yet neglected step towards preventing antimicrobial resistance.

**Electronic supplementary material:**

The online version of this article (10.1186/s40249-018-0459-7) contains supplementary material, which is available to authorized users.

## Multilingual abstract

Please see Additional file 1 for translations of the abstract into the five official working languages of the United Nations.

## Background

Poverty, or “the pronounced deprivation of well-being”, still affected an estimated 767 million people worldwide in 2013 despite remarkable efforts [1]. Such deprivation can refer to income (i.e., low individual or household income) and non-income dimensions such as limited education, unemployment or precarious employment, inadequate housing conditions, insufficient access to healthcare, clean water and sanitation [2, 3]. The reductions in income poverty achieved by the Millennium Development Goals (MDGs) have however not been coupled with significant improvements in other non-income dimensions.

Poverty greatly increases the risk of contracting infectious diseases, with clinical outcomes further aggravated by lacking access to healthcare. Poor waste and sanitation, non-potable drinking water, housing overcrowding [4] and inadequate nutrition [5] are all linked to risk of and recovery from infectious diseases. Furthermore, the interconnection between the different dimensions of poverty with infectious diseases across countries with varying economic status has also been well established [6–9].

In this context, addressing the leading global public health threat of antimicrobial resistance (AMR) is critical as it is invariably connected to infectious diseases, but also a major threat to achievement of the sustainability goals. In the last decade, global consumption of antibiotics in human health increased by 40%, primarily in emerging economies [10, 11]. Inappropriate use of antibiotics facilitated by unrestricted access without prescription in many countries [12], self-medicating practices [13], or using old and leftover antibiotics for a new health problem [14] further exacerbates AMR. The scale of AMR-attributable mortality is estimated at ~ 700 000 deaths per year worldwide [15] and raises issues particularly relevant to the most vulnerable citizens in society when achieving balance of ‘excess’ versus ‘access’ to antibiotic treatment [16].

The association between different dimensions of poverty and antibiotic exposure is not limited to low- and middle-income countries (LMICs). In Sweden, experiences of economic stress and low educational level increased the odds of taking antibiotics [17], and in the United Kingdom (UK), higher antibiotic prescribing rates in primary care have been observed in deprived areas [18, 19]. Several studies have shown increased antibiotic use among high socio-economic groups, particularly in countries where private health insurance is the norm [20]. Thus, the evidence on the link between poverty and antimicrobial exposure, and subsequent AMR remains unclear.

However, there is lack of evidence illustrating the relationship between the dimensions of poverty and the prevalence of AMR. Identifying poverty-related factors associated with antimicrobial resistant infections would inform policies beyond inappropriate antimicrobial use and targeting interventions aimed at high risk groups, helping policymakers allocate resources to address dimensions of poverty that influence AMR. Such approach would have the potential for benefit not only on individual and public level health outcomes – but also from the economic perspective of the health system and societal level.

This paper investigates the association of AMR and poverty by identifying the dimensions of poverty that are potential factors in the acquisition of antimicrobial-resistant organisms in humans.

## Methods

### Search strategy

This systematic review followed the Preferred Reporting Items for Systematic Reviews and Meta-Analyses (PRISMA) guidelines [21]. We searched the PubMed, Ovid, MEDLINE, EMBASE, Scopus, CINAHL, PsychINFO, EBSCO, HMIC, and Web of Science databases in October 2016. No date limits were placed on the published articles. Search strings were tailored to each database. Where Medical Subject Headings (MeSH) terms and EMBASE headings were applicable, the main root of the term was used. Combinations of the following search terms were used: “poverty”, “socioeconomic factor”, “socioeconomic status”, “income”, “antimicrobial resistance”, “antibiotic resistance”, “antiviral resistance”, “antifungal resistance”, “housing”, “residence characteristics”, “living conditions”, “educational status”, “health literacy”, “employment”. Terms where truncated as necessary.

### Inclusion and exclusion criteria

We included prospective and retrospective studies that reported on income or non-income dimensions of poverty as variables and their influence on the acquisition of antimicrobial-resistant organisms. Cohort, case-control, and cross-sectional studies were included. We considered colonization and infection on any anatomical site and organ system, among all age groups in any country. We included all published literature written or translated in English, except ecological studies, reviews, commentaries, and editorials.

Studies that used surrogates for antimicrobial resistance such as antimicrobial consumption, dispensing and prescribing were excluded. We also excluded studies exclusively exploring the relationship between poverty and HIV, tuberculosis and malaria, as reviews on these areas have already been published [22]. These studies generally explored the impact of poverty on non-adherence to antiretroviral therapy, the financial burden of complying with treatment, or the link between a single dimension of poverty such as educational attainment or housing conditions and the acquisition of these infections.

### Study screening and selection

**Fig. 1:**
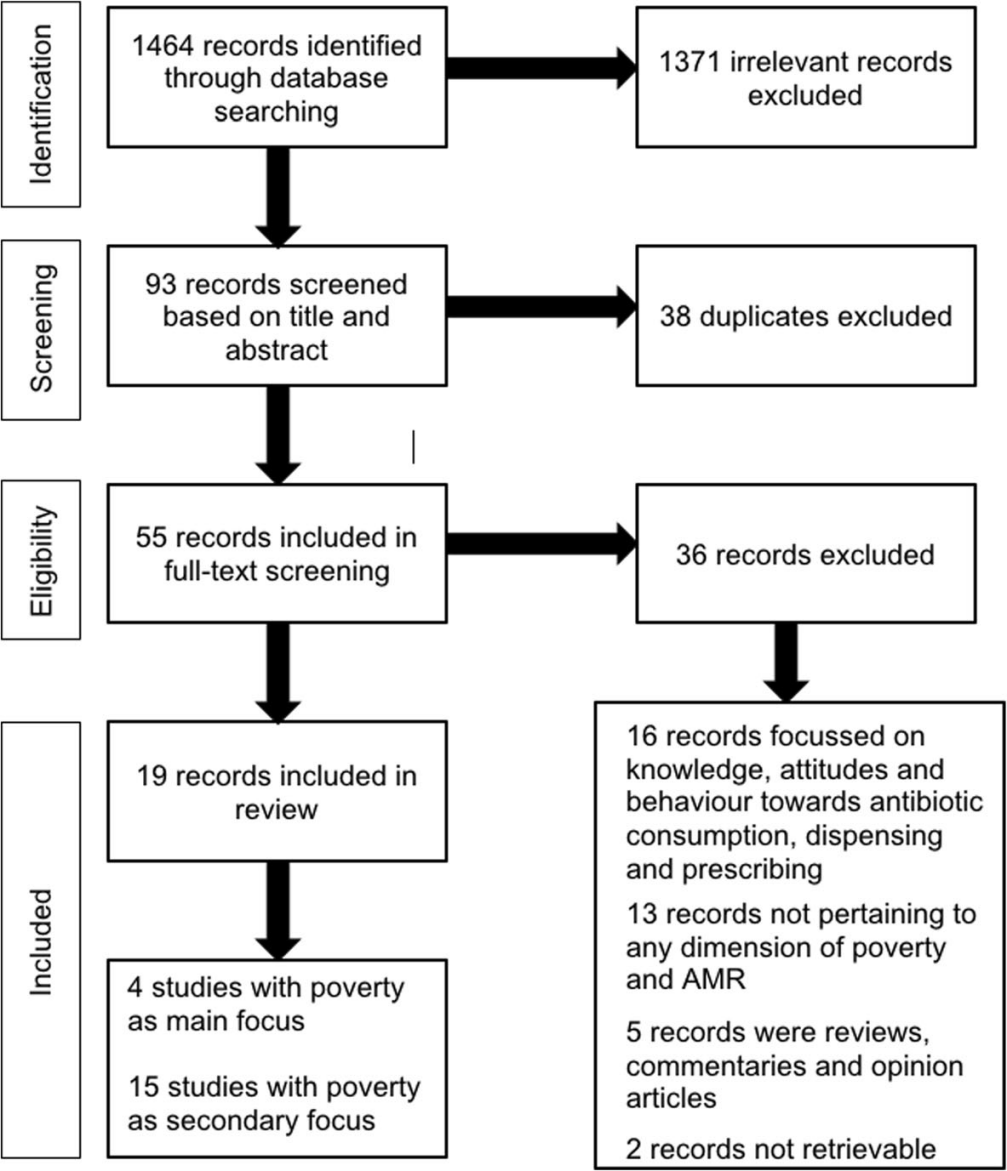
Study flowchart

Study screening and selection was aided by software Rayan (https://rayyan.qcri.org/) (Fig. 1). Two researchers (VM & VA) independently and concurrently screened each title and abstract, with any discrepancies about the inclusion/exclusion of articles mediated by a third researcher (ECS).

### Data extraction

A standardised electronic data extraction form was completed with pertinent information from each source. The following data were extracted: country of study, type of study, study population, sample size, dimension of poverty, measurement level of poverty, type of resistance and organism, colonization or infection reported, and the association between the dimensions of poverty referred in the study to AMR.

### Study quality assessment

The integrated quality criteria for review of multiple study designs (ICROMS) [23] was used to conduct quality assessment of the papers selected. ICROMS is a comprehensive and practical tool in appraising a range of studies to be included in systematic reviews, particularly public health-related studies. The tool outlines the quality criteria specific to a study design and it determines the robustness and the relevance of the study to the review question using a decision matrix and scoring system. As recommended, studies were not excluded based on their appraised quality.

### Categorisation of studies according to poverty dimensions

The studies selected were grouped around the following dimensions of poverty: a) housing and living conditions, b) income and income inequality, c) education level, d) water and sanitation and e) social deprivation.

### Results

Due to the heterogeneity of settings, populations and dimensions of poverty explored, the results are reported in narrative format. Table 1 shows the summary of studies discussed, including their ICROMS scores. Of the 1464 articles retrieved, we excluded 1371, full-text screened 55 and finally included 19 in the review. The included studies were published mainly in infectious diseases, microbiology, and epidemiology journals, with a number of articles from general medical and other non-infectious disease specialist sources. Included articles were published from 1998 to 2015. Twelve studies were conducted in high-income countries (HICs), predominantly in the United States of America (USA) followed by the UK.

**Table 1:** Identified studies, by dimension of poverty and setting

Low-and middle-income countries		Dimension of poverty	High income countries	
Association with antimicrobial resistance			Association with antimicrobial resistance	
Negative	Positive		Positive	Negative
Duerink 2007 (Indonesia) [31] Seidman 2009 (India) [36] Kristiansson 2009 (Peru) [37]	Lestari 2010 (Indonesia) [24]	Housing and living conditions	Young 2004 (USA) [26] Bratu 2006 (USA) [56] Jourdain 2010 (Belgium) [44]	Nilsson 2005 (Sweden) [25]
Duerink 2007 (Indonesia) [31]	Lestari 2010 (Indonesia) [24]	Income and income inequality	Chen 1998 (USA) [27] McMullen 2009 (USA) [28] Henig 2015 (Israel) [29]	No studies found
Duerink 2007 (Indonesia) [31] Seidman 2009 (India) [36] Kristiansson 2009 (Peru) [37] Boyanova 2009 (Bulgaria) [38]	Trecker 2014 (China) [32]	Education level	Huang 2004 (USA) [33] Garcia-Rey 2004 (Spain) [34]	Nilsson 2005 (Sweden) [25]
No studies found	Seidman 2009 (India) [36] Souza 2009 (Brazil) [39]	Water and Sanitation	No studies found	No studies found
No studies found	No studies found	Social depriation	Bagger 2004 (UK) [40] Nomamiukor 2015 (UK) [35]	Parsons 2001 (UK) [41]

### Housing and living conditions

Crowding, homelessness, and living environment have been associated with antimicrobial resistant isolates in individuals in the community as well as hospital patients (Table 2). 3995 community residents and hospital patients were enrolled between 2001 and 2002 in a study in Indonesia to determine nasal carriage of *Staphylococcus aureus* and determinants of such carriage, including demographic, socioeconomic, health and antimicrobial exposure variables [24]. The carriage prevalence was 9.1% (362/3995), and overcrowding (defined as households with more than eight persons) was positively associated with carriage (*OR* = 4.5, 95% *CI*: 1.4–15.1) in the community.

**Table 2:** Studies identified exploring relation between antimicrobial resistance, housing and living conditions

Authors and year	Country	Study design	Participants	Population	Measurement level of poverty	Microorganism	Colonisation / infection	Association with AMR	ICROMS score
Jourdain et al. 2010	Belgium	Cohort	1347	Healthy children from 11 pre-schools in Brussels	Household	Resistant *Streptococcus pneumoniae*	Colonisation	Positive	20
Lestari et al. 2010 [24]	Indonesia	Cohort	3995	Patients from 2 hospitals and 3 primary health centres in 2 cities in Java	Household	Resistant *Staphylococcus aureus*	Colonisation	Positive	24
Duerink et al. 2007 [31]	Indonesia	Retrospective data analysis	3275	Patients from 2 hospitals and 3 primary health centres in 2 cities in Java	Household	Resistant *Escherichia coli*	Colonisation	Negative	Not applicable
Bratu et al. 2006 [56]	USA	Cohort	1316	Patients from 15 hospitals in Brooklyn, New York	Community/Neighbourhood (provincial-wide); official census data	Community-Associated MRSA	Not specified	Positive	18
Nilsson and Laurell 2005	Swede	Retrospective data analysis	766	Residents from Malmö	Community/Neighbourhood (city-wide)	Penicillin non-susceptible *Streptococcus pneumoniae*	Not specified	Negative	Not Applicable
Bagger et al. 2004 [44]	UK	Cohort	1739	UK residents undergoing isolated coronary artery bypass graft in London	Community/Neighbourhood	MRSA	Infection	Positive	21
Young et al. 2004 [26]	USA	Retrospective data analysis	837	Patients from 1 specialist clinic in San Francisco	Community/Neighbourhood	MRSA	Infection	Positive	Not Applicable
Parsons et al. 2001 [41]	UK	Cohort	1064	Patients from 1 hospital in Sheffield	Community/Neighbourhood	Resistant *Helicobacter pylori*	Not specified	Negative	17

*ICROMS* Integrated quality criteria for review of multiple study designs, *MRSA* Methicillin-resistant *Staphylococcus aureus*

Nilsson et al. [25] estimated the relation between penicillin-non-susceptible *Streptococcus pneumoniae* (defined by the authors as MIC ≥0.5 mg/ml for penicillin) and socio-economic factors including household crowding in 19 residential areas in Malmo, Sweden. The incidence of PNSP cases was not associated to any of the socioeconomic variables and only with antibiotic prescribing (*r* = 0.614, *P* < 0.01).

In the USA, lack of housing or homelessness was identified as a risk factor for methicillin-resistant *S. aureus* (MRSA) infection in medically underserved patients with soft tissue infections in a retrospective analysis conducted in 2004 [26]. This review of attendees to a specialised center in San Francisco, California included more than 7700 surgical procedures over 3 years. A subanalysis of the first 666 patients with positive *S. aureus* and MRSA culture suggested that transmission of infection was reported to occur in the community, with MRSA associated with injection drug use (*OR* = 1.8; *P* = 0.003) and homelessness (*OR* = 1.5; *P* = 0.03).

### Income and income inequality

Evidence on the relationship between low income and antimicrobial resistance in HICs has evolved (Table 3). In 1998, Chen et al. reported a negative association between income and *S. pneumoniae* resistance in the USA, using population-based surveillance for invasive pneumococcal disease linked with neighbourhood-level censal data such as age, race and address [27]. The association, however, seemed to exist only amongst Caucasians and not African-American citizens.

**Table 3:** Studies identified exploring relation between antimicrobial resistance, income and income inequality

Authors and year	Country	Study design	Participants	Population	Measurement level of poverty	Microorganism	Colonisation /infection	Association with AMR	ICROMS score
Henig et al. 2015 [29]	Israel	Matched Case-Control Study	6998	Patients at largest heath maintenance organisation in Israel	Community	Carbapenem-resistant *Acinetobacter baumannii*	Both	Positive	Not applicable
Lestari et al. 2010 [24]	Indonesia	Cohort	3995	Patients from 2 hospitals and 3 primary health centres in 2 cities in Java	Household	Resistant *Staphylococcus aureus*	Colonisation	Positive	24
McMullen et al. 2009 [28]	USA	Retrospective data analysis	10 530	Adult patients from hospital in St. Louis	Community	Community-Associated MRSA	Not specified	Positive	Not applicable
Duerink et al. 2007 [31]	Indonesia	Retrospective data analysis	3275	Patients from 2 hospitals and 3 primary health centres in 2 cities in Java	Household	Resistant *Escherichia coli*	Colonisation	Negative	Not applicable
Chen et al. 1998 [27]	USA	Retrospective data analysis	716	Isolates from 33 laboratories in Atlanta, Georgia	Community	Drug-resistant invasive pneumococcal infections	Infection	Negative	Not applicable
Trecker et al. 2014 [32]	China	Cross-sectional	384	Patients from hospital in Shanghai	Individual	Resistant *Neisseria gonorrheae*	Infection	Positive	Not applicable

*AMR* Antimicrobial resistance, *ICROMS* Integrated quality criteria for review of multiple study designs, *MRSA* Methicillin-resistant *Staphylococcus aureus*

More recent evidence in the same country established a link between MRSA infection and low income [28]. The median income of the postcode of each person with infection was used as a proxy, with low income defined as a median annual income below 25 000 USD. The authors however acknowledged that such categorisation of income was a limitation.

In Israel, individuals with low socioeconomic status had almost twice the risk of carbapenem-resistant *Acinetobacter baumannii* (CRAB) colonisation and bacteraemia compared to counterparts in high socioeconomic strata (*OR* = 2.18, 95% *CI*: 1.02–5). The population-based case-control study conducted between 2007 and 2012 matched 1190 hospital patients with CRAB and compared them to other 1190 hospitals patients without *Acinetobacter* infection but similar risk factors [29]. However, socioeconomic status in this investigation was not exclusively related to income but also based on the patient’s outpatient clinic affiliation. In Israel, such affiliation determines the type of healthcare coverage received [30], which may be indicative of income. The authors explained their results by either healthcare access disparities including healthcare coverage or by patients’ characteristics.

The relationship between income and antimicrobial-resistant infections in LMICs presents more challenges. Unlike evidence from HICs that generally included income as a single variable, researchers from LMICs frequently reported it bundled with and at least one other dimension of poverty. Two Indonesian studies presented contradictory associations between income and AMR. Duerink et al. [31] did not find any association between low income and *Escherichia coli* resistance, whilst other researchers [24] suggested that low income was a determinant factor for the carriage of MRSA. Both studies had the same population of interest and classified income level as either below or above the poverty line.

In China, males in the lowest income category were more likely have antibiotic-resistant *Neisseria gonorrhoea* compared with other individuals in the middle-income category [32]. However, the findings in this study were limited by the small sample size and the limited choice of variables included in the multilevel regression model.

### Education level

In HICs, lack of education seems positively associated with the acquisition of resistant infections (Table 4). In the USA, low parental educational attainment significantly predicted carriage of antibiotic-resistant pneumonia in young children, as reported in a community study with 710 children that evaluated variables such as household size, household income and limited plumbing facilities [33].

**Table 4:** Studies identified exploring relation between antimicrobial resistance and education level

Authors and year	Country	Study design	Participants	Population	Measurement level of poverty	Microorganism	Colonisation /infection	Association with AMR	ICROMS score
Nomamiukor et al. 2015 [35]	UK	Retrospective data analysis	2775	Primary healthcare patients in 2 cities in Northwest England	Community	Resistant *Escherichia coli*	Not specified	Positive	Not applicable
Trecker et al. 2014 [32]	China	Cross-sectional	384	Patients from hospital in Shanghai	Individual	Resistant *Neisseria gonorrheae*	Infection	Positive	Not applicable
Boyanova et al. 2009 [38]	Bulgaria	Cohort	266	Untreated *Helicobacter pylori* patients from 4 hospitals in Sofia	Individual	Resistant *H. pylori*	Not specified	Negative	19
Kristiansson et al. 2009 [37]	Peru	Cross-sectional	nearly 1600	2 rural communities in Amazonian Peru	Household	Resistant *E. coli*	Not specified	Negative	Not applicable
Seidman et al. 2009 [6]	India	Cross-sectional	120	2 rural villages in Tamil Nadu	Household	Resistant *E. coli*	Colonisation	Positive	Not applicable
Duerink et al. 2007 [31]	Indonesia	Retrospective data analysis	3275	Patients from 2 hospitals and 3 primary health centres in 2 cities in Java	Household	Resistant *E. coli*	Colonisation	Negative	Not applicable
Nilsson and Laurell 2005	Sweden	Retrospective data analysis	766	Residents from Malmö	Community	Penicillin non-susceptible *Streptococcus pneumoniae*	Not specified	Negative	Not applicable
Garcia-Rey et al. 2004 [34]	Spain	Retrospective data analysis	2726	Isolates from laboratories from 15 provinces in Spain; Official provincial population demographic data from the National Statistics System	Community	Resistant *S. pneumoniae*	Not specified	Positive	Not applicable
Huang et al. 2004 [33]	USA	Cohort study	742	Young children from 16 Massachusetts communities	Community	Resistant *S. pneumoniae*	Both	Positive	20

*AMR* Antimicrobial resistance, *ICROMS* Integrated quality criteria for review of multiple study designs

Similar findings were obtained in Spain [34] when analysing 2726 bacterial isolates from clinical samples submitted to the national reference laboratory, with antibiotic-resistant *S. pneumoniae* more frequently identified in adults with less than primary school education. A recent UK study also established that community-level indicators of low adult education, skills and training, along with poor living conditions, were two key domains impacting on the prevalence of cefuroxime- and nitrofurantoin-resistant *E. coli* [35].

Not all evidence in HICs, however, has established an association between low educational level and antimicrobial resistance. In Sweden, adults with less than upper secondary school education were no more likely to develop antibiotic-resistant pneumonia, although total prescribing of antibiotics was positively correlated with per capita income (*r* = 0.597, *P* < 0.05) [25].

In LMICs, on the other hand, the educational level of the population did not seem to have an impact on the rates of antimicrobial resistance. In Indonesia, incomplete primary school education was not associated to carriage of resistant *E. coli* [31]*.* Similar results have been observed in rural India [36] and the Peruvian Amazon [37]. Research on resistant *Helicobacter pylori* in Bulgaria reported a negative association with educational level [38]. In that study, where 266 consecutive *H. pylori* strains isolated from untreated patients were evaluated in 2004–2008, education was stratified as either ‘higher education’ or ‘other’ without detailed definitions. Unlike the other studies in LMICs, adult males with less than primary school education in China were more likely to be diagnosed with antibiotic-resistant gonorrhoea [32].

### Water and sanitation

Clean water, safe and effective sanitation systems are central to preventing diarrhoeal diseases. Therefore, the burden of disease associated with poor water and sanitation often has a greater effect on those living in poverty in LMICs. Two papers explored the association between water and sanitation and antimicrobial resistance in LMICs, whilst no studies in HICs focused on these variables (Table 5).

**Table 5:** Studies identified exploring relation between antimicrobial resistance, water and sanitation

Authors and year	Country	Study design	Participants	Population	Measurement level of poverty	Microorganism	Colonisation/ infection	Association with AMR	ICROMS score
Souza et al. 2009 [39]	Brazil	Case-control	79	Children 5–10 years old from the Colinas D’Oeste slum	Household	Resistant *Escherichia coli*	Infection	Positive	Not applicable
Seidman et al. 2009 [36]	India	Cross-sectional	120	2 rural villages in Tamil Nadu	Community	Resistant *Escherichia coli*	Colonisation	Positive	Not applicable

*AMR* Antimicrobial resistance, *ICROMS* Integrated quality criteria for review of multiple study designs

Researchers in Brazil reported the wide distribution of potentially pathogenic *E. coli* strains among asymptomatic children of low socioeconomic status [39]. In this study, 79 school-age children between 5 and 10 years living in a slum with no sewage system, occasional water supply and infrequent domestic waste collection, were matched with 35 children who attended a private school of the same city. Resistance to either sulphonamides (52%) or cotrimoxazole (35%) was more frequent in cases from the slum (65%) than the control group (16%).

In India, the type of household water purification method in two rural villages was associated with carriage of resistant *E. coli* in primary school children [36]. Such rates were determined from stool samples from primary school children, and attendance to the local primary school was used a substitute for the type of water purification method used at these children’s homes.

### Social deprivation

When investigating whether post-operative infection with MRSA was associated with the socioeconomic background of patients undergoing coronary artery bypass grafting in a London hospital over a 5-year period, patient postcodes were linked according to social deprivation demonstrating that citizens from most deprived neighbourhoods had a seven-fold higher infection rate than those from the least deprived (Table 6) [40]. At the time of the study it was not possible to determine whether the results reflected a greater proportion of MRSA carriers living in the most deprived areas, or increased patient susceptibility to MRSA infection among those more socially deprived.

**Table 6:** Studies identified exploring relation between antimicrobial resistance, social deprivation

Authors and year	Country	Study design	Participants	Population	Measurement level of poverty	Microorganism	Colonisation /infection	Association with AMR	ICROMS score
Duerink et al. 2007 [31]	Indonesia	Retrospective data analysis	3275	Patients from 2 hospitals and 3 primary health centres in 2 cities in Java	Household	Resistant *Escherichia coli*	Colonisation	Negative	Not applicable
Nilsson and Laurell 2005	Sweden	Retrospective data analysis	766	Residents from Malmö	Community	Penicillin non-susceptible *Staphylococcus pneumoniae*	Not specified	Negative	Not applicable
Bagger et al. 2004 [40]	UK	Cohort	1739	UK residents undergoing isolated coronary artery bypass graft in 1 London hospital	Community	MRSA	Infection	Positive	21
Parsons et al. 2001 [41]	UK	Cohort	1064	Patients from 1 hospital in Sheffield	Community	Resistant *Helicobacter pylori*	Not specified	Negative	17

*AMR* Antimicrobial resistance, *ICROMS* Integrated quality criteria for review of multiple study designs, *MRSA* Methicillin-resistant *Staphylococcus aureus*

Another study used surveillance data of urinary *E. coli* isolates collected from patients attending primary care services in England with suspected urinary tract infection in 2010–2012 [35]. The multilevel logistic regression models estimated that participants’ living environment and residency in the most deprived areas was associated with increased odds of antibiotic resistance in the isolates (*OR =* 1.33 [95% *CI*: 1.07–1.75] –2.47 [95% *CI*: 1.08–5.66]) for different antibiotics.

In the UK again, a negative association was found when exploring the potential link between socioeconomic status as measured by the Jarman score, and resistance to metronidazole (*P* = 0.95) or a macrolide (*P* = 0.31) in a cohort of 1064 patients undergoing endoscopy between 1994 and 1999 [41].

## Discussion

This review examined the association between dimensions of poverty and carriage or infection with antimicrobial-resistant microorganisms. The variety of poverty dimensions identified were reported based on socioeconomic status, generally ascertained via proxy indicators, geographical or census information. The limited number of papers identified had low to moderate methodological quality and were predominantly conducted in high income countries. Despite the heterogeneity of the studies, the results suggest an association between a range of dimensions of poverty and antimicrobial-resistant infections across all countries.

We identified a total of 19 studies, yet only four explicitly examined the association of poverty and AMR, suggesting a need for further studies. Level of education, low income, housing conditions, water and sanitation were positively associated with AMR. Among the studies in HICs, positive associations were identified between housing conditions, lack of education and low income, while in turn water and sanitation were positively associated with AMR in LMICs. Although there were differences in the strength of the relation across the dimensions of poverty, intermediate determinants such as level of income, level of education and housing conditions exhibited a relatively consistent association with AMR. The methodologies employed did not however allow for analysis of the mediating factors which may explain these observations. For example, the extent to which factors influencing health-seeking behaviours are individual capacity issues and those that arise from structural aspects, such as disparities in insurance packages available.

In terms of pathogens, the material dimensions of poverty (housing, living conditions, social deprivation) seemed to be associated with the carriage or infection with resistant *S. aureus*. Perhaps this is not surprising, considering characteristics of the organism such as persistence in clinical and community environments [42], with obvious importance to mitigate its transmission and acquisition [43]. On the other hand, in the case of infections with *S. pneumoniae*, the differing results obtained by Jouirdain et a. (2010) [44] and Nilsson et al. [25] may reflect increased exposure to antibiotics, even in a high-income country as Belgium, and as also seen in similar settings [45].

Although the association of education with resistance in *E. coli* appeared to be equally present and absent, such discrepancy may again reflect the diverse effect of material dimensions of poverty such as housing conditions including sanitation facilities –likely to be the case in the study by Seidman et al. [36] or the effect of increased exposure to antimicrobials [35] or lack of exposure to them [31, 37].

The variability of results obtained in the *E. coli* studies exemplifies well the careful equilibrium that societies and health systems have to achieve and sustain between reducing the likelihood of infection with resistant organisms due to lack of material resources versus similar infections resulting from excessive use of antibiotics. Future studies considering the effect of socioeconomic determinants on infections and drug-resistant organisms may benefit from increasing the objective as well as subjective precision used to establish individual, household and community poverty. Additionally, it may be extremely useful to examine the effect of rapid transitions away from deprivation (for example, due to gentrification [46]) or towards poverty (for instance, as a result of the recent financial crash and subsequent austerity measures [47]).

Colonisation or infection with resistant pathogens may affect any group in society over lifetime, however vulnerability due to poverty may increase such risk. Socio-economic deprivation in housing, environment and work affects however the relationship between health and ill health from utero to older age [48]. The World Health Organization’s report on the social determinants of health emphasised actions that eradicate poverty and enhance opportunities for health and well-being [49]. Health inequalities place individuals or populations already vulnerable at a further disadvantage in terms of infections. Consultations fees or the price of antimicrobials may push the poorest citizens to likely suboptimal antimicrobials. Additionally, these same patients may be offered consultations of poorer quality in terms of information and involvement, when compared to other groups [50]. The information shortcomings and disempowerment, coupled with health literacy deficits fuelled by shared dimensions of poverty [51] are likely to impact on perceptions and understanding of antimicrobials [52] and reinforce subsequent inappropriate use.

Globally, sustaining finance and leadership have been identified as key to achieving access while preventing excess of antimicrobial use [53]. Whilst good access to and utilisation of health services is associated with equitable health care, for low and middle income countries the issues of clean water and sanitation, lack of infrastructure and access to vaccines which result in repeated infections and antimicrobial use, possibly contributing to the burden of antimicrobial resistance in these settings [54], will have to be resolved in parallel to empowering citizens to improve health seeking behaviours and attitudes towards antimicrobial use.

This review has several limitations. We focussed on published peer-reviewed journal articles and therefore excluded grey literature. The studies extracted from this review originated from eleven countries, therefore limiting generalisation to other countries, LMICs particularly. Considering a likely ascertainment bias (i.e., antimicrobial susceptibility testing and reporting much less frequently available in LMICs) and the geographical gradient for some pathogens (e.g. *Acinetobacter*) [55], such under-representation of LMICs may be even more threatening to any generalisability considerations. Methodological difficulties inherent to the use of aggregate scores to measure poverty create difficulty in distinguishing the dimensions of poverty with the greatest impact on AMR. Further, most studies included were of low to moderate quality, although the ICROMS tool may appear to be applicable to only a few study designs. Finally, publication bias may limit the conclusions drawn.

## Conclusions

These studies, despite methodological and qualitative limitations, elicited the influence of dimensions of poverty compounding AMR. Assumptions are often made of the pathway leading dispossessed citizens with low income, or living in poor communities, to develop antibiotic resistant infections. Whilst the association between colonization or infection with drug-resistant organisms and socioeconomic factors, particularly poor living conditions, poor sanitation, low level education or health literacy and low income requires further research, addressing the social determinants of poverty across national policies worldwide may remain a crucial yet neglected step towards preventing antimicrobial resistance.

## Additional file

Additional file 1: Multilingual abstracts in the five official working languages of the United Nations. (PDF 840 kb)

## Abbreviations

AMR: Antimicrobial resistance
*CI*: Confidence interval
CRAB: Carbapenem-resistant *Acinetobacter baumannii*
HIC: High-income country
HIV: Human immunodeficiency virus
ICROMS: Integrated quality criteria for review of multiple study designs
LMICS: Low- and middle-income country
MDG: Millennium Development Goals
MRSA: Methicillin-resistant *Staphylococcus aureus*
*OR*: Odd ratio
PNSP: Penicillin non-susceptible *Streptococcus pneumoniae*
PRISMA: Preferred Reporting Items for Systematic Reviews and Meta-Analyses
UK: United Kingdom
USA: United States of America
