# Supplementary material for: Investigating the impact of poverty on colonization and infection with drug-resistant organisms in humans: a systematic review
Source: Infect Dis Poverty. 2018 Aug 17;7:76. doi: 10.1186/s40249-018-0459-7 (PMC6097281; doi:10.1186/s40249-018-0459-7)
Supplement: Supplementary file 1 — Multilingual abstracts in the five official working languages of the United Nations. (PDF 840 kb) [file 40249_2018_459_MOESM1_ESM.pdf]

Translation of the abstract into the five official working languages of the United Nations

التحقيق في تأثير الفقر على العدوى والإصابة بالكائنات المقاومة للعقاقير في البشر: مراجعة منهجية

ڤيڤيان أليڤيدزا - ڤيكتور ماريانو - راحيلة أحمد - إسميتا تشاراني - تيموثي راوسون - أليسون هولمز - إنريك كاسترو-سانشيز

ملخص

خلفية: يزيد الفقر من خطر التقاط الأمراض المعدية وبالتالي التعرض للمضادات الحيوية. ومع ذلك ، هناك نقص في الأدلة على العلاقة بين أبعاد الدخل وأبعاد غير الدخل المتعلقة بالفقر ومقاومة مضادات الميكروبات. تدارس مثل هذه العلاقة من شأنه أن يعزز من التدخلات للإشراف المضادة للميكروبات.

النص الرئيسي: تم إجراء مراجعة منهجية بعد إعداد بنود القواعد الإرشادية لـ "التقارير المفضلة للمراجعات المنهجية والتحليل البَعدي" (PRISMA). في أكتوبر 2016 تم البحث في قواعد بيانات مواقع PubMed و Ovid و MEDLINE و EMBASE و Scopus و CINAHL و PsychINFO و EBSCO و HMIC وشبكة العلوم "Web of Science". تم تجديد الدراسات المستقبلية والمرجعية التي أبلغت عن أبعاد الدخل أو غير الدخل للفقر وتأثيرها على الاستعمار أو العدوى بالكائنات المقاومة للميكروبات. تم تقييم جودة الدراسة بمعايير الجودة المتكاملة لاستعراض أداة تصاميم الدراسة المتعددة (ICROMS). تمت مراجعة تسع عشرة مقالة. ارتبط الازدحام والتشرد بمقاومة مضادات الميكروبات لدى مرضى المجتمع المحلي والمستشفيات. في الدول ذات الدخل المرتفع، يرتبط الدخل المنخفض بمناهضة الجرثومة العقيدة الرئوية والباكتيريا الراكدة البومانية ومعدل إصابة أعلى بسبعة أضعاف. في الدول ذات الدخل المنخفض كانت النتائج حول هذه العلاقة متناقضة. نقص التعليم كان مرتبطًا بمقاومة الجرثومة العقدية الرئوية والباكتيريا الإشريكية. ورقتان بحثتا في العلاقة بين الماء والتعزيز الصحي ومقاومة مضادات الميكروبات في المحيط منخفض الدخل.

الاستنتاجات: برغم القيود المنهجية، تشير النتائج إلى كون معالجة المحددات الاجتماعية للفقر في جميع أنحاء العالم لا تزال خطوة حاسمة نحو منع مقاومة مضادات الميكروبات، ولكنها خطوة مهملة.

Translated from English version into Arabic by Mohamed Hamdi, proofread by Free bird, through

TRANSLATORS WITHOUT BORDERS

贫困对人类耐药菌定植和感染的影响：一项系统综述

Vivian Alividza, Victor Mariano, Raheelah Ahmad, Esmita Charani, Timothy Rawson, Alison Holmes, Enrique Castro-Sánchez

摘要

引言：贫困增加了感染传染病的风险，进而增加了暴露于抗生素的风险。然而，目前尚无证据表明抗生素耐药性与贫困的收入和非收入维度之间存在相关性。

方法：本文根据系统综述和荟萃分析优先报告的条目（PRISMA）指南进行系统综述。2016 年 10 月在 PubMed、Ovid、MEDLINE、EMBASE、Scopus、CINAHL、PsychINFO、EBSCO、HMIC 和 Web of Science 数据库，检索关于贫困的收入和非收入维度及其对耐药菌定植或感染造成影响的前瞻性和回顾性研究。采用综合质量标准审查多项研究设计(ICROMS)工具进行评估。

结果：审查了 19 篇文章。拥挤和无家可归与社区和医院患者的抗生素耐药性有关。在高收入国家，低收入与肺炎链球菌和鲍曼不动杆菌耐药性相关，感染率高出 7 倍。在低收入国家，关于这一关系的调查结果是相互矛盾的。缺乏教育与耐药肺炎链球菌和大肠杆菌有关。两篇论文探讨了低收入环境中水和环境卫生与抗生素耐药性之间的关系。

结论：尽管存在方法上的局限性，但结果表明，解决全球贫困的社会决定因素仍然是防止抗生素耐药性的关键步骤。

Translated from English version into Chinese by Translated by Pei Wang, edited by Pin Yang

CHINESE CENTER FOR DISEASE CONTROL AND PREVENTION
NATIONAL INSTITUTE OF PARASITIC DISEASES

Étude de l’impact de la pauvreté sur la colonisation et l’infection par des organismes résistant à l’effet des médicaments chez les humains: revue systématique

Vivian Alividza, Victor Mariano, Raheelah Ahmad, Esmita Charani, Timothy Rawson, Alison Holmes, Enrique Castro-Sánchez

Résumé

Contexte: La pauvreté accroît le risque de contracter des maladies infectieuses et, par voie de conséquence, celui d'une exposition aux antibiotiques. Il n'existe cependant aucune donnée démontrant de façon probante une relation entre les dimensions de la pauvreté liées ou non aux revenus, d'une part, et la résistance antimicrobienne d'autre part. L'étude de cette relation serait pourtant intéressante dans l'optique d'une utilisation raisonnée des agents antimicrobiens.

Méthodes: Une revue systématique a été réalisée suivant les lignes directrices PRISMA (items à privilégier pour le compte rendu des revues systématiques et des méta-analyses). Nous avons effectué des recherches dans les bases de données PubMed, Ovid, MEDLINE, EMBASE, Scopus, CINAHL, PsychINFO, EBSCO, HMIC et Web of Science en octobre 2016. Nous avons collecté des études prospectives et rétrospectives portant sur les dimensions de la pauvreté liées ou non aux revenus et leur influence sur la colonisation ou l'infection par des organismes résistants aux antimicrobiens. La qualité de ces études a été évaluée à l'aide de l'outil ICROMS (critères intégrés de qualité pour la revue d'études multiples).

Résultats: Nous avons inclus dix-neuf articles dans notre revue. La surpopulation et le sans-abrisme étaient associés à la résistance aux antimicrobiens en milieu communautaire et hospitalier. Dans les pays à revenus élevés, un faible revenu était associé à la résistance de *Streptococcus pneumoniae* et d'*Acinetobacter baumannii* et à un taux d'infection sept fois plus élevé. Dans les pays à bas revenus, les résultats concernant cette association étaient contradictoires. Le manque d'éducation était lié à la résistance de *S. pneumoniae* et d'*Escherichia coli*. Deux rapports ont exploré la relation entre l'eau et l'assainissement, d'une part, et la résistance aux antimicrobiens, d'autre part, dans les milieux à faibles revenus.

Conclusions: Malgré les limites méthodologiques des études, les résultats suggèrent que la prise en compte des déterminants sociaux de la pauvreté dans le monde reste une étape cruciale mais négligée dans la prévention de la résistance aux antimicrobiens.

Translated from English version into French by Paustelita, proofread by Suzanne Assenat, through

TRANSLATORS
WITHOUT BORDERS

Исследование взаимосвязи бедности и уровня инфицирования устойчивыми к лекарственным препаратам микроорганизмами: систематический обзор

Вивиан Алвидза’ Виктор Мариано’ Рахила Ахмад’ Эсмита Чарани’ Тимоти Росон’ Алисон Холмс’ Энрике Кастро-Санчес

Аннотация

Справочная информация. Бедность повышает риск заражения инфекционными заболеваниями, а следовательно, увеличивает применение антибиотиков. Тем не менее имеется недостаточно доказательств наличия взаимосвязи между устойчивостью к противомикробным препаратам и проявлениями бедности, как связанными, так и не связанными с доходами. Исследование указанных взаимосвязей позволит укрепить управление противомикробными мероприятиями.

Методы. Систематический обзор производился в соответствии с рекомендациями, заложенными в положениях руководства по предпочтительным позициям отчетности для систематических обзоров и метаанализов (PRISMA). Поиск проводился в октябре 2016 года по базам данных PubMed, Ovid, MEDLINE, EMBASE, Scopus, CINAHL, PsychINFO, EBSCO, HMIC и Web of Science. Были отобраны исследования по перспективному и ретроспективному анализу, освещающие как связанные, так и не связанные с доходами проявления бедности, а также их влияние на уровень инфицирования устойчивыми к лекарственным препаратам микроорганизмами. Оценка качества исследований производилась при помощи алгоритма комплексных критериев оценки уровня качества для обзора множественных моделей исследований (ICROMS).

Результаты. Обзор проводился по девятнадцати научным статьям. Перенаселенность и бездомность ассоциировались с устойчивостью к противомикробным препаратам в сообществах,

а также среди стационарных больных. В странах с высоким уровнем дохода низкий уровень дохода ассоциировался с устойчивостью бактерий *Streptococcus pneumoniae* и *Acinetobacter baumannii* , а также с семикратным повышением уровня инфицирования. Выводы по данной взаимосвязи в странах с низким уровнем дохода оказались противоречивыми. Низкий уровень образования связывался с устойчивостью к антибиотикам штаммов *S. pneumoniae* и *Escherichia coli*. В двух научных работах исследовалась взаимосвязь между доступом к водоснабжению и канализации и устойчивостью к противомикробным препаратам в условиях низкого дохода.
Выводы. Несмотря на ограничения используемой методологии, результаты свидетельствуют о том, что устранение социальных факторов бедности в мировом контексте по-прежнему представляет собой ключевой, но не получающий должного внимания, шаг на пути к предотвращению устойчивости к противомикробным препаратам.

Translated from English version into Russian by Liudmila Tomanek, proofread by Anna Romanenko, through

TRANSLATORS
WITHOUT BORDERS

El impacto de la pobreza en humanos colonizados e infectados por organismos resistentes a los medicamentos: una revisión sistemática

Vivian Alividza, Victor Mariano, Raheelah Ahmad, Esmita Charani, Timothy Rawson, Alison Holmes, Enrique Castro-Sánchez

Resumen
Antecedentes: La pobreza aumenta el riesgo de contraer enfermedades infecciosas y, por lo tanto, la exposición a antibióticos. Aún así, carecemos de evidencia sobre la relación entre las dimensiones de la pobreza en cuanto a los ingresos o a la falta de ellos, y la resistencia antimicrobiana. Investigar tal relación fortalecería las intervenciones de la administración antimicrobiana.
Métodos: Se realizó una revisión sistemática según las pautas *Ítems de Información Preferidos para Evaluaciones Sistemáticas y Metanálisis (PRISMA)*. Se realizaron búsquedas en las bases de datos de PubMed, Ovid, MEDLINE, EMBASE, Scopus, CINAHL, PsychINFO, EBSCO, HMIC y Web of Science en octubre de 2016. Se obtuvieron estudios prospectivos y retrospectivos que informaban sobre las dimensiones de la pobreza en cuanto a los ingresos o a la falta de ellos, y su influencia en la colonización o infección con organismos resistentes a los antimicrobianos. La calidad del estudio se evaluó con la herramienta decriterios integrados de calidad para la revisión de múltiples diseños de estudio (ICROMS).
Resultados: Se revisaron diecinueve artículos. Se asoció el hacinamiento y la falta de vivienda con la resistencia a los antimicrobianos en pacientes de la comunidad y del hospital. En los países de altos ingresos, los bajos ingresos se asociaron con el *estreptococo la neumonía* y *la* resistencia al Acinetobacter baumannii y a una tasa de infección siete veces mayor. En países de bajos ingresos, los hallazgos sobre esta relación fueron contradictorios. La falta de educación se vinculó con *S. pneumoniae* y *Escherichia coli* resistentes. Dos artículos exploraron la relación entre el agua y el saneamiento y la resistencia a los antimicrobianos en entornos de bajos ingresos.
Conclusiones: A pesar de las limitaciones metodológicas, los resultados sugieren que abordar los determinantes sociales de la pobreza en todo el mundo sigue siendo un paso crucial pero desatendido para prevenir la resistencia a los antimicrobianos.

Translated from English version into Spanish by Guadalupe Barua, proofread by Maria Paula Gorgone, through

TRANSLATORS
WITHOUT BORDERS
